# Nonpolymeric Citramide-Based Kinetic Hydrate Inhibitors: Good Performance with Just Six Alkylamide Groups

**DOI:** 10.1021/acsomega.2c00448

**Published:** 2022-04-13

**Authors:** Radhakanta Ghosh, Malcolm A. Kelland

**Affiliations:** † Department of Mathematics and Natural Science, Faculty of Science and Technology, University of Stavanger, Stavanger N-4036, Norway; ‡ Department of Chemistry, Bioscience and Environmental Engineering, University of Stavanger, Stavanger N-4036, Norway

## Abstract

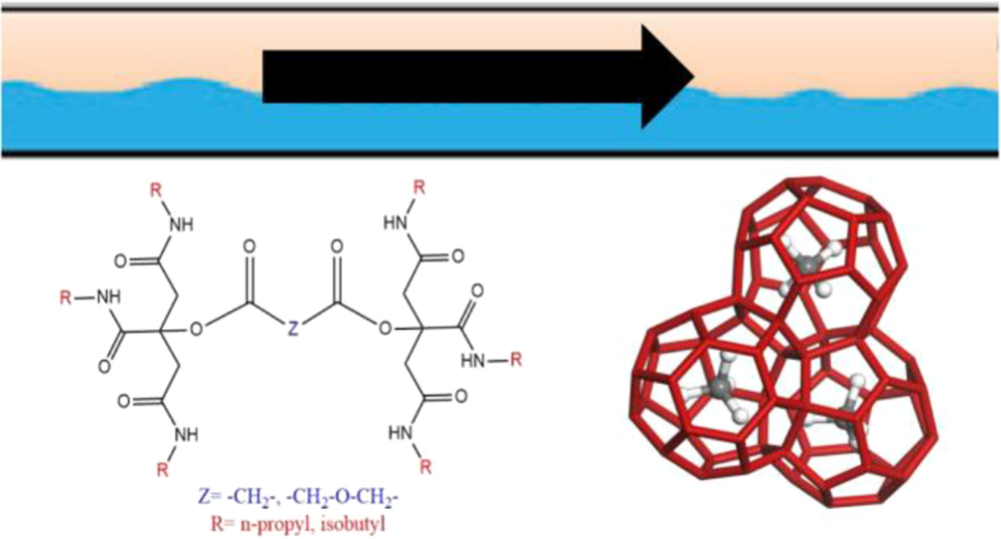

The use of kinetic hydrate inhibitors (KHIs) is a well-known method for preventing gas hydrate formation in oil and gas production flow lines. The main ingredient in KHI formulations is one or more polymers with amphiphilic groups. Here, we report a series of citramide-based nonpolymeric KHIs. The KHI performance of these citramide derivatives has been studied using a synthetic natural gas mixture (forming structure II hydrate as the thermodynamically preferred phase) in slow constant cooling (ca. 1 °C/h starting from 20.5 °C) high-pressure (76 bar) rocking cell experiments. Isobutyl-substituted alkyl chains in the mono/bis(trialkyl citric acid) amide derivative gave better KHI performance than *n*-propyl-substituted citramide derivatives. Moreover, biscitramides with six alkylamide functional groups gave better performance than the equivalent monocitramides with three alkylamide groups. A solution of 2500 ppm of bis(tributyl citric acid) amide gave an average gas hydrate onset temperature (*T*_o_) of 8.4 °C compared to 8.9 °C for a low molecular weight *N*-vinyl pyrrolidone/*N*-vinyl caprolactam 1:1 copolymer. For the bis(tributyl citric acid) amide, addition of liquid hydrocarbon (*n*-decane) lowered further the average *T*_o_ value to 6.2 °C, although this is at least partly due to lowering of the hydrate equilibrium temperature. This study demonstrates that good KHI performance can be obtained from molecules with as little as six amphiphilic alkylamide groups.

## 1 Introduction

Under high pressure and low temperature, free water molecules in the presence of small gas molecules such as methane, propane, butane, nitrogen, and carbon dioxide can form a thermodynamically stable crystalline solid water lattice structure, called gas hydrates. Solid crystalline water cages are held together through hydrogen bonding and stabilized by van der Waals forces between the trapped guest gas molecules and the water cages. In gas and oil pipelines, the potential for the formation of gas hydrate plugging is a major flow assurance issue. Low dosage hydrate inhibitors (LDHIs) are a well-established technology for the chemical prevention of these plugs. The strategy with LDHIs is generally a two-step process, either delaying hydrate formation kinetically or controlling the formation and deposition of hydrate crystals. The first method led to the development of kinetic hydrate inhibitors (KHIs), and the second method led to antiagglomerants (AAs).^1−6^

The main ingredient in an injected KHI formulation is one or more water-soluble polymers. They are usually dosed into the produced water between 0.1 and 2.0 wt % together with the carrier solvent(s), synergists, and other production chemicals. Most current commercial KHI polymers are polyamides such as homopolymers and copolymers of *N*-vinyl lactams or *N*-isopropylmethacrylamide, as well as polyesteramides such as hyperbranched poly(esteramide)s or polyester pyroglutamates.^3−6,10,11^ However, polymers with other hydrophilic groups besides amides have been shown to have good KHI performance, especially polyamine oxides.^12−14^ This may be due to the strong hydrogen bonding afforded by both amides and amine oxides. KHIs are able to delay gas hydrate crystal nucleation and crystal growth depending on the subcooling, residence time, and a range of other factors including salinity, pressure, liquid hydrocarbon, and other production chemicals. There is also evidence that KHIs can totally inhibit hydrate crystal growth up to a certain subcooling level.^7−9^

**Figure 1:**
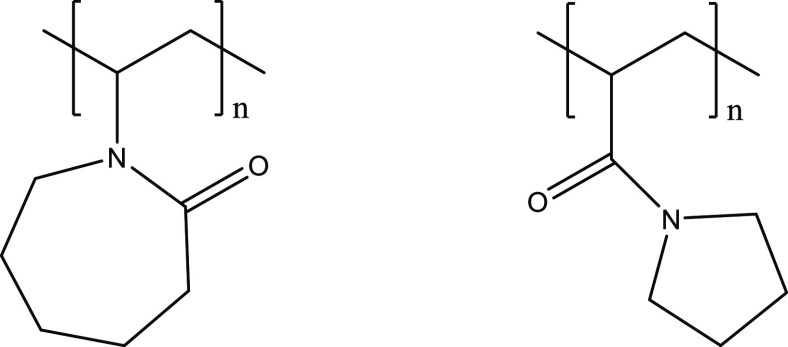
Poly(*N*-vinyl caprolactam) (PVCap, left) and polyacryloylpyrrolidine (PAP, right).

**Figure 2:**
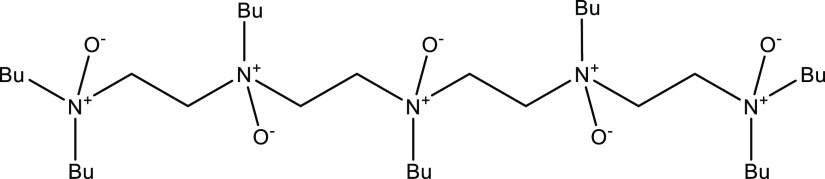
Heptabutylated amine oxide of tetraethylenepentamine.

The polymer molecular weight (*M*_W_) and *M*_W_ distribution are important factors that affect the KHI performance. For unimodal *M*_W_ distributions, several experimental studies suggest that very low *M*_W_ polymers (oligomers) are preferred for gas hydrate kinetic inhibition, possibly as low as 4–8 monomer units. Early work with polyvinylcaprolactam (PVCap) found that the highest subcooling performance was obtained with a polymer *M*_W_ of 900 g/mol, and the next best *M*_W_ was 1300 g/mol with decreasing performance as the *M*_W_ increased (Figure 1).^12^ A more recent study on PVCap showed that *M*_n_ (number average *M*_W_) values as low as 449 and 508 g/mol gave good KHI performance.^13^ This represents about 3–4 monomer units if the gel permeation chromatography analysis at these values is considered reliable. Polyacryloylpyrrolidine (PAP) polymers gave poor KHI performance for *M*_n_ = 300 g/mol (about three monomer units) but good performance at 870 g/mol (about seven monomer units) (Figure 2).^14^ Another study on PVCap suggests that for unimodal *M*_W_ distributions, a polymer with a narrow distribution is the best.^15^

However, there is evidence that a bimodal distribution of *M*_W_s can give better KHI performance.^16^ Preferably, the majority of the product has low *M*_W_ and a minor portion has higher *M*_W_. That is why some KHI polymer formulations are a mixture of two polymer products with varying *M*_W_ distributions. A rationale for this was recently proposed based on the Gibbs–Thomson effect.^17^ Based on this theory, a continuum of *M*_W_s with a majority at low *M*_W_ would be even better. Nonpolymeric molecules that have multiple KHI-active functional groups have also been investigated. For example, butylated polyethyleneamine oxides with 3–5 N atoms and 5 to 7 butyl groups, respectively, have been shown to perform well as KHIs on both structure I (sI) and structure II (sII) gas hydrates (Figure 2).^18,19^

**Figure 3:**
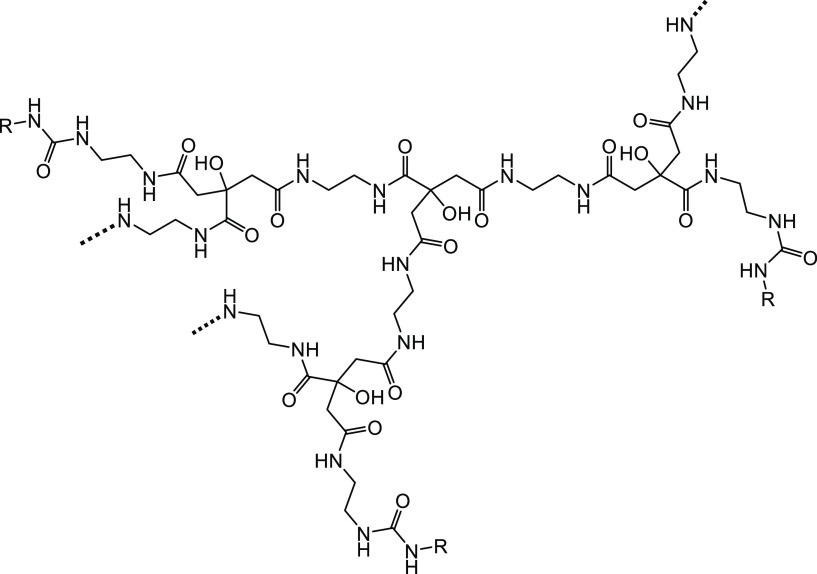
N-terminal NH_2_ groups in poly(ethylene citramide)s. R = isopropyl, *n*-butyl, or cyclohexyl groups.

We have previously investigated poly(ethylene citramide)s and shown that a low *M*_W_ polymer with pendant *N*-cyclohexyl groups gave good KHI performance on an sII hydrate-forming gas mixture (Figure 3).^20^

**Figure 4:**
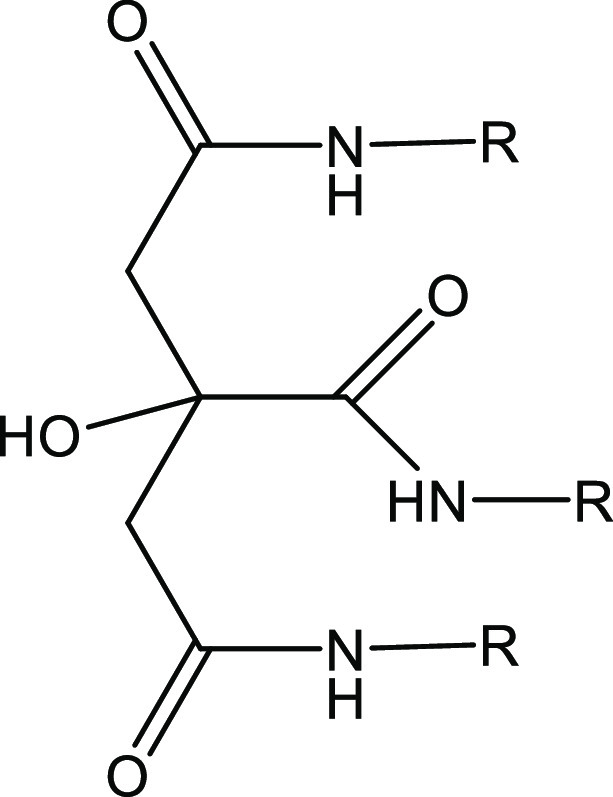
Trialkylcitramides.

Trialkylcitramides have also been investigated as KHIs (Figure 4).^21^ Trialkylcitramides (or citric acid trialkylamides) are made by reaction of primary amines with citric acid esters.^21−24^ None of the trialkylcitramides gave very good performance, with *n*-propyl being the best. Either butylated derivatives were not water-soluble or only partial ester-to-amide conversion occurred, probably for steric reasons.

**Figure 5:**
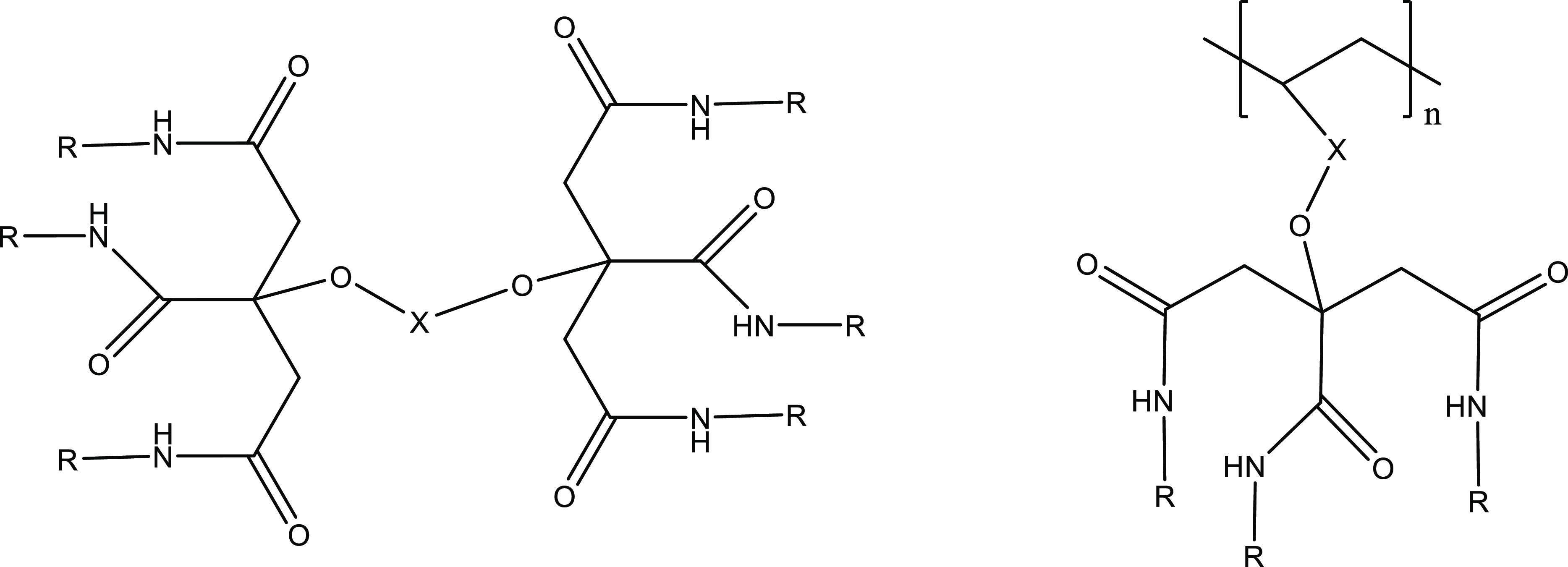
Bis(trialkyl citric acid) amide with spacer groups −C(*O*)CH_2_C(O)– or −C(*O*)CH_2_OCH_2_C(O)– (left), and a vinyl polymer with pendant trialkylcitramide groups, where *n* = 8–9.

It occurred to us that the mediocre KHI performance of the trialkylcitramides could be improved by increasing the number of alkylamide groups in the molecule. Two structural variations seemed worth trying (Figure 5):

1. Attaching the trialkylcitramide to a short polyvinyl polymer backbone via the hydroxyl group.
2. Making a bis(trialkyl citric acid) amide with six alkylamide groups, by esterifying two trialkylcitramide molecules with a diacid.

Here, we report our findings in carrying out these two structural modifications. We demonstrate that the KHI performance of trialkylcitramides can indeed be significantly improved, particularly by careful use of the second method.

## 2 Experimental Section

### 2.1 Chemicals

Maleic anhydride, *n*-pentylamine, and 2,2′-oxydiacetyl dichloride were purchased from TCI Europe. Triethyl citrate, *n*-propylamine, and isobutyl amine were purchased from Merck. Malonyl dichloride was obtained from Alfa Aesar. Solvents like *n*-decane, iso- and *n*-butyl glycol ether (nBGE), dichloromethane (DCM), methanol, and *o*-xylene were supplied by VWR chemicals. CDCl_3_ was purchased from Cambridge Isotope Laboratories, Inc. All the chemicals were used without any further purification. Synthesis of polymaleic anhydrides (PMA, *M*_W_ = 800, dispersity 3.8) was carried out according to the literature, except that *o*-xylene was used here as the solvent instead of toluene.^25^ An *N*-vinyl pyrrolidone/*N*-vinyl caprolactam 1:1 copolymer (VP:VCap 1:1) (*M*_n_ 2000–4000 g/mol, 53.8 wt % in water) was obtained from BASF.

### 2.2 Spectral Analysis

^1^H spectra were obtained with a Bruker Ascend NMR 400 MHz spectrometer in tubes with a 5 mm external diameter. CDCl_3_ was used as a solvent.

### 2.3 Synthesis

#### 2.3.1 Synthesis of 2-Hydroxy-*N*^1^,*N*^2^,*N*^3^-trialkyl-1,2,3-tricarboxamide (Trialkyl Citramide)

All the trialkyl citramides were synthesized by reaction of corresponding alkyl amine with triethylcitrate. The detailed synthesis protocol has been described previously.^22^ In brief, triethyl citrate (1 equiv), alkyl amine (3.3 equiv), and methanol were taken in a 100 mL round-bottom flask and kept for stirring at room temperature for 3 days. Then, additional 3.3 equiv of alkyl amine was injected into the reaction mixture and kept for stirring at room temperature for one more day. The mixture was then dried in rota-vacuum at 70 °C for 2 h. The resulting pale-yellow/white solid was washed with diethyl ether 3 times and collected via filtration and dried at 60 °C overnight to give a white powder as a final product.

#### 2.3.2 Synthesis of 2-Hydroxy-*N*^1^,*N*^2^,*N*^3^-tripropylpropane-1,2,3-tricarboxamide (tri-*n*-propylcitramide) (TnPrCAm)

TnPrCAm was synthesized by reaction of *n*-propylamine with triethylcitrate (yield = 85%). ^1^H NMR (400 MHz, CDCl_3_) δ ppm: 7.18 (1H, -N**H**-), 6.95 (2H, -N**H**-), 6.72 (1H, -O**H**), 3.17 (6H, -NH-C**H_2_**-), 2.74–2.70 (2H, -C**H_2_**-CO-), 2.56–2.52 (2H, -C**H_2_**-CO-), 1.5 (6H, -CH_2_-C**H_2_**-CH_3_), 0.89 (9H, -CH_2_-C**H_3_**).

#### 2.3.3 Synthesis of 2-Hydroxy-*N*^1^,*N*^2^,*N*^3^-triisobutylpropane-1,2,3-tricarboxamide (tri-iso-Butylcitramide) (TiBuCAm)

TiBuCAm was synthesized by reaction of isobutylamine with triethylcitrate (yield = 78%). ^1^H NMR (400 MHz, CDCl_3_) δ ppm: 7.21 (1H, -N**H**-), 7.04 (2H, -N**H**-), 6.96 (1H, -O**H**), 3.03 (6H, -NH-C**H_2_**-), 2.78–2.75 (2H, -C**H_2_**-CO-), 2.60–2.55 (2H, -C**H_2_**-CO-), 1.74 (3H, -C**H**-(CH_3_)_2_), 0.89 (18H, -CH-(C**H_3_**)_2_).

#### 2.3.4 Synthesis of 2-Hydroxy-*N*^1^,*N*^2^,*N*^3^-tripentylpropane-1,2,3-tricarboxamide (tri-*n*-pentylcitramide) (TiPeCAm)

TiPeCAm was synthesized by reaction of *n*-pentylamine with triethylcitrate (yield = 55%). ^1^H NMR (400 MHz, CDCl_3_) δ ppm: 7.1 (1H, -N**H**-), 7.0 (2H, -N**H**-), 6.9 (1H, -O**H**), 3.2 (6H, -NH-C**H_2_**-), 2.73–2.69 (2H, -C**H_2_**-CO-), 2.55–2.52 (2H, -C**H_2_**-CO-), 1.58 (3H, -C**H**-(CH_3_)_2_), 1.37 (6H, -C**H_2_**-CH-(CH_3_)_2_), 0.89 (18H, -CH-(C**H_3_**)_2_).

#### 2.3.5 Synthesis of Bis(trialkyl citric acid) Amide

Bis(trialkyl citric acid) amides were synthesized by reaction of trialkyl citramides with the relative aliphatic acid-dichloride according to the following general procedure.^26^ In detail, in a 100 mL round-bottom flask with a magnetic stirrer, a solution of respective aliphatic acid-dichloride (2.0 mmol) in DCM (10 mL) was added slowly over 30 min to a DCM (20 mL) solution of trialkyl citramide (4.4 mmol) and NEt_3_ (1.1 mL, 8 mmol) at 0 °C. After stirring at a low temperature for 1 h, the reaction mixture was kept for stirring overnight at room temperature. Then, the reaction mixture was filtered and washed with a solution of 5% HCl (2 × 50 mL), then with a saturated solution of sodium bicarbonate (2 × 50 mL), and finally with deionized water (50 mL). The organic phase was passed through anhydrous Na_2_SO_4_, and then, the solvent was evaporated under reduced pressure to get the final product.

#### 2.3.6 Synthesis of 1,3-Malonic acid bis(tripropyl citric acid) amide (Malonyl-TnPrCAm)

Malonyl-TnPrCAm was synthesized by reaction of TnPrCAm with malonyl dichloride (yield = 55%). ^1^H NMR (400 MHz, CDCl_3_) δ ppm: 7.2 (2H, -N**H**-), 6.9 (4H, -N**H**-), 3.37 (2H, -CO-C**H_2_**-CO-), 3.2 (12H, -NH-C**H_2_**-), 2.73–2.69 (4H, -C**H_2_**-CO-), 2.59–2.55 (4H, -C**H_2_**-CO-), 1.50 (12H, -CH_2_-C**H_2_**-CH_3_), 0.91 (18H, -CH_2_-C**H_3_**).

#### 2.3.7 Synthesis of 2,2′-Oxydiacetic acid bis(tripropyl citric acid) amide (Oxydiacetyl-TnPrCAm)

Oxydiacetyl-TnPrCAm was synthesized by reaction of TnPrCAm with oxydiacetyl dichloride (yield = 52%). ^1^H NMR (400 MHz, CDCl_3_) δ ppm: 7.15 (2H, -N**H**-), 6.88 (4H, -N**H**-), 4.21 (4H, -C**H_2_**-CO-C**H_2_**-), 3.17 (12H, -NH-C**H_2_**-), 2.75–2.72 (4H, -C**H_2_**-CO-), 2.57–2.54 (4H, -C**H_2_**-CO-), 1.52 (12H, -CH_2_-C**H_2_**-CH_3_), 0.90 (18H, -CH_2_-C**H_3_**).

#### 2.3.8 Synthesis of 1,3-Malonic acid bis(triisobutyl citric acid) amide (Malonyl-TiBuCAm)

Malonyl-TiBuCAm was synthesized by reaction of TiBuCAm with malonyl dichloride (yield = 50%). ^1^H NMR (400 MHz, CDCl_3_) δ ppm: 7.22 (2H, -N**H**-), 6.95 (4H, -N**H**-), 3.4 (2H, CO-C**H_2_**-CO-), 3.05 (12H, -NH-C**H_2_**-), 2.86–2.82 (4H, -C**H_2_**-CO-), 2.70–2.66 (4H, -C**H_2_**-CO-), 1.78 (6H, -C**H**-(CH_3_)_2_), 0.91 (36H, -CH-(C**H_3_**)_2_).

#### 2.3.9 Synthesis of 2,2′-oxydiacetic acid bis(triisobutyl citric acid) amide (Oxydiacetyl-TiBuCAm)

Oxydiacetyl-TiBuCAm was synthesized by reaction of TiBuCAm with oxydiacetyl dichloride (yield = 45%). ^1^H NMR (400 MHz, CDCl_3_) δ ppm: 7.22 (2H, -N**H**-), 6.95 (4H, -N**H**-), 4.21 (4H, -C**H_2_**-CO-C**H_2_**-), 3.05 (12H, -NH-C**H_2_**-), 2.79–2.75 (4H, -C**H_2_**-CO-), 2.60–2.56 (4H, -C**H_2_**-CO-), 1.76 (6H, -C**H**-(CH_3_)_2_), 0.90 (36H, -CH-(C**H_3_**)_2_).

#### 2.3.10 Synthesis of Polymaleic Trialkylcitramide Esters

In general, one equivalent of trialkyl citramide was used for each repeating unit of polymaleic anhydride. Polymaleic tripropylcitramide ester (PMA-TnPrCAm) was synthesized by mixing PMA and TnPrCAm under melt (100 °C) and vacuum conditions overnight in the presence of the acid catalyst. Polymaleic triisobutylcitramide ester (PMA-TiBuCAm) was made in dimethoxyethane at 85° for 2 days by mixing PMA and TiBuCAm. Polymaleic-(tripropylcitramide-triisobutylcitramide) ester (PMA-TnPrCAm-TiBuCAm (1:1)) was made in acetone by stirring at room temperature for 3 days by reacting PMA with a 1:1 mixture of TnPrCAm and TiBuCAm. As this is an addition reaction, the *M*_W_s of polymaleic trialkylcitramide esters can be calculated as the sum of the *M*_W_s of the PMA and trialkylcitramide.

### 2.4 Cloud Point (*T*_Cl_) Determination

A 2500 ppm aqueous solution of each citramide derivative was heated slowly (ca. 5 °C/min), and the first sign of clouding of the solution at a certain temperature was denoted as the cloud point. Any solution that was already opaque at room temperature was first kept in a cooling room at 0–5 °C overnight before heating. Each measurement was repeated at least three times to check the reproducibility of the cloud point.

### 2.5 Kinetic Hydrate Inhibitor (KHI) Performance Tests in High-Pressure Rocking Cells

**Figure 6:**
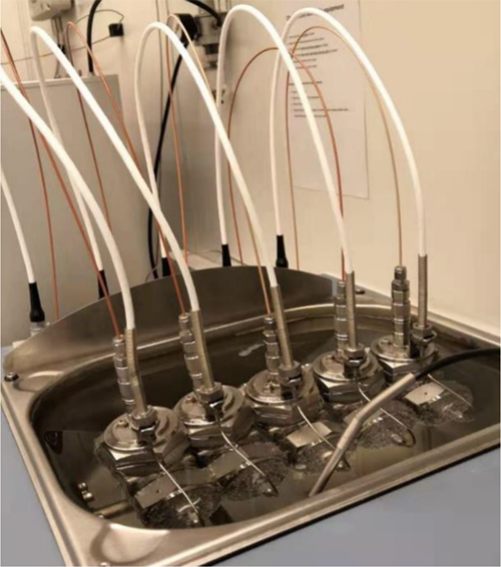
KHI test equipment showing the steel rocking cells placed in a cooling bath. Reproduced from Ghosh, R. and Kelland, M. A., Pushing the Known Performance Envelope of Kinetic Hydrate Inhibitors—Powerful Synergy of Trialkylamine Oxides with Acrylamide-Based Polymers, Energy Fuels 2022, 36, 1, 341–349. Copyright 2022. American Chemical Society.

A rig with five high-pressure steel rocking cells was used for carrying out the KHI performance ranking of the new molecules. Each cell has a volume of 40 mL and a steel ball for agitation. The rig was supplied by PSL Systemtechnik GmbH, Germany, but the cells were constructed by Swafas, Norway (Figure 6). As in most of our KHI studies, we used a synthetic natural gas (SNG) mixture in order to compare results with a host of previous KHI studies. The SNG gives an sII hydrate as the thermodynamically stable phase (Table 1). Also, for comparative purposes we used our standard slow constant cooling (SCC) test method.^27^ The pressure was approximately 76 bar at the start of each SCC experiment. At this pressure, an average equilibrium temperature (*T*_eq_) of 20.2 °C ± 0.05 °C was obtained from five repeat tests by laboratory slow dissociation experiments in the same rocking cells.^20^ This agrees very well with a *T*_eq_ value of 20.5 °C calculated using Calsep’s PVTSim software.

**Table 1:** Composition of the Synthetic Natural Gas (SNG) Mixture

component	mol %
nitrogen	0.11
*n*-butane	0.72
isobutane	1.65
propane	5.00
CO_2_	1.82
ethane	10.3
methane	80.4

The SCC test procedure was carried out as follows:

1. First, 20.0 mL of aqueous test solution was added to each steel cell, i.e., half the cell volume.
2. Air was removed from the cells by alternate vacuum and filling with 1–5 bar SNG gas.
3. The cells were filled with a 76 bar SNG mixture at room temperature, 20.5 °C.
4. The cells were cooled from 20.5 to 2 °C at a rate of 1 °C/h while rocking.
5. Temperature and pressure data for each cell were collected from the sensors and recorded throughout the whole cooling procedure.

**Figure 7:**
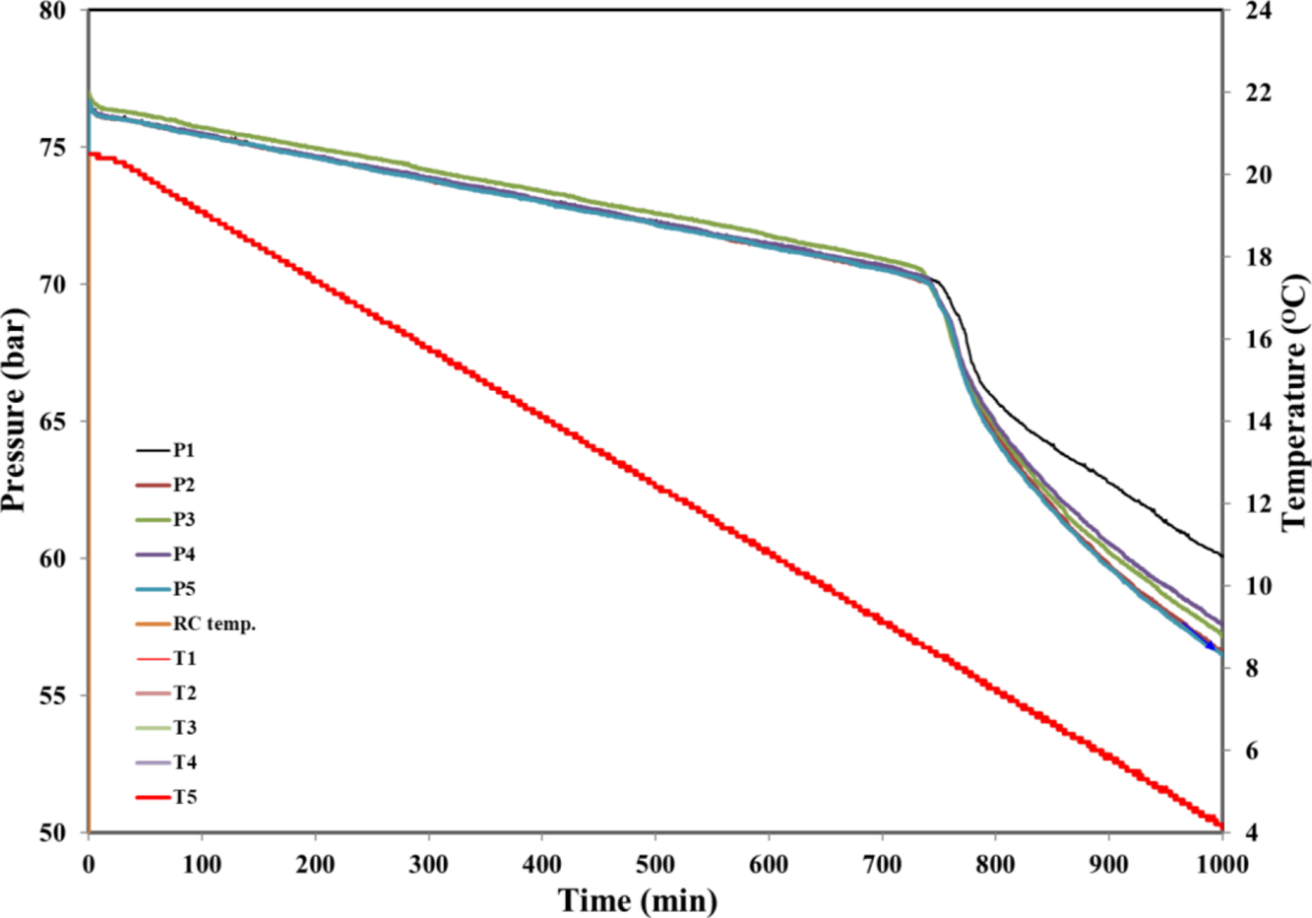
Typical pressure–time and temperature–time graphs of all five cells obtained from a standard constant cooling KHI experiment. This example is for 2500 ppm of oxydiacetyl-TiBuCAm.

**Figure 8:**
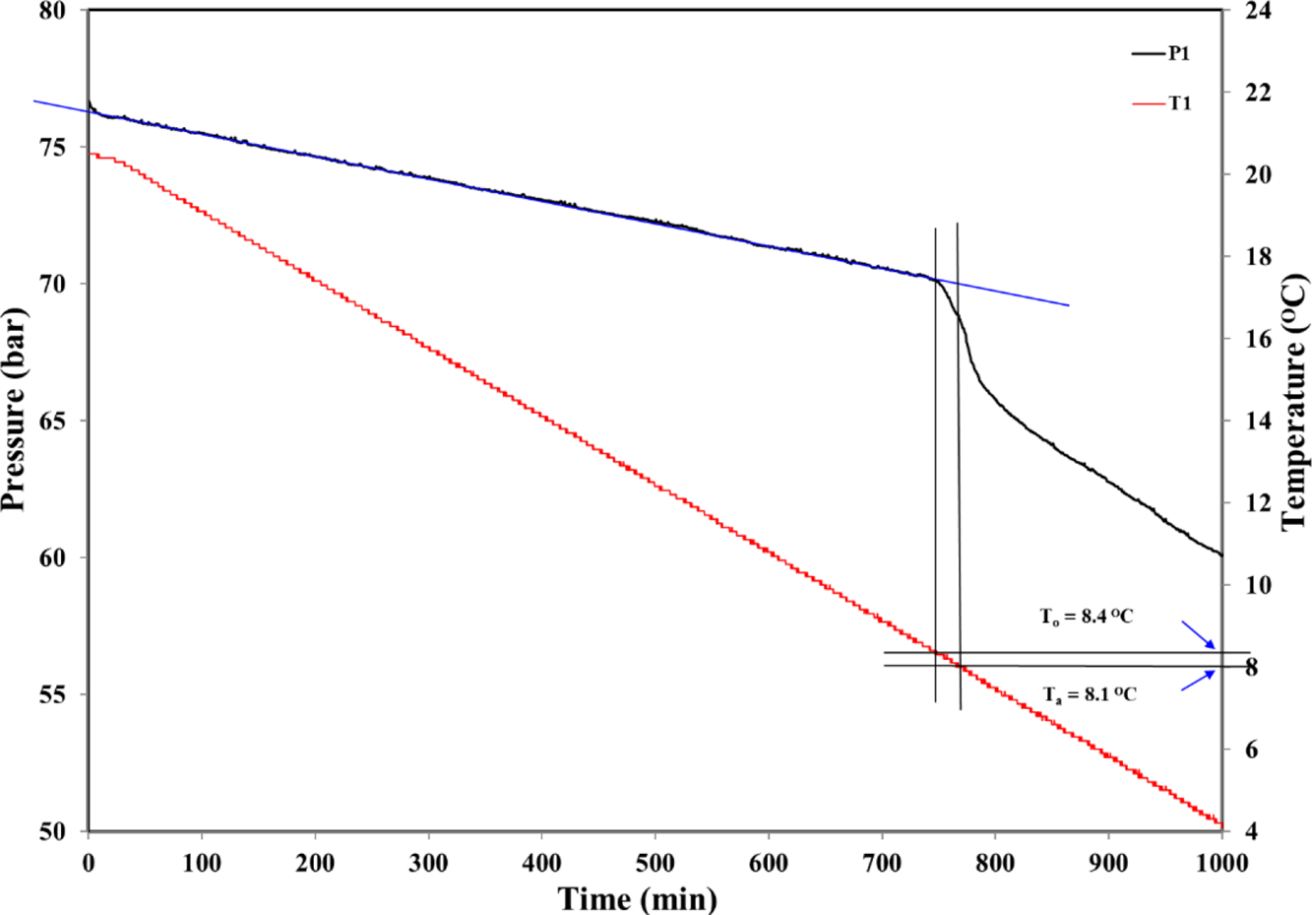
Example of *T*_o_ and *T*_a_ calculation in a standard constant cooling KHI experiment. This example is for 2500 ppm of oxydiacetyl-TiBuCAm.

Figure 7 shows a typical graph of pressure and temperature vs time using the same chemical in all five cells. Two parameters are determined from these data, the gas hydrate onset temperature (*T*_o_) and the gas hydrate rapid formation temperature (*T*_a_). Figure 8 illustrates how this is done for one cell. The small pressure drop at the very start of the graph is due to some SNG mixture dissolving in the aqueous phase. After this, the pressure drop is linear until hydrate formation is first detected at a *T*_o_ of 8.4 °C, where the pressure line deviates. Nucleation may have occurred somewhat earlier but is not detectable. *T*_a_ is the first temperature at which we observe the maximum rate of gas hydrate formation for the experiment.

At 8.1 °C (*T*_a_), the first fastest pressure drop rate occurs. In this case, the rate of hydrate growth is fast shortly after *T*_o_. When *T*_o_ is not very low, this indicates that the KHI is not very good at arresting crystal growth. The degrees of scattering in *T*_o_ values (≤20%) and *T*_a_ values (≤15%) are due to the stochastic nature of gas hydrate formation and are as expected from previous studies.^28^ The scattering still allows for a rough ranking of the performance of the KHI samples as long as sufficient tests are carried out for a statistically significant difference using a *t*-test. Depending on the variation in average *T*_o_ between samples, 5–10 tests suffices in most cases to get a significant difference at the 95% confidence level (*p* < 0.05).^29^

## 3 Results and Discussion

**Figure 9:**
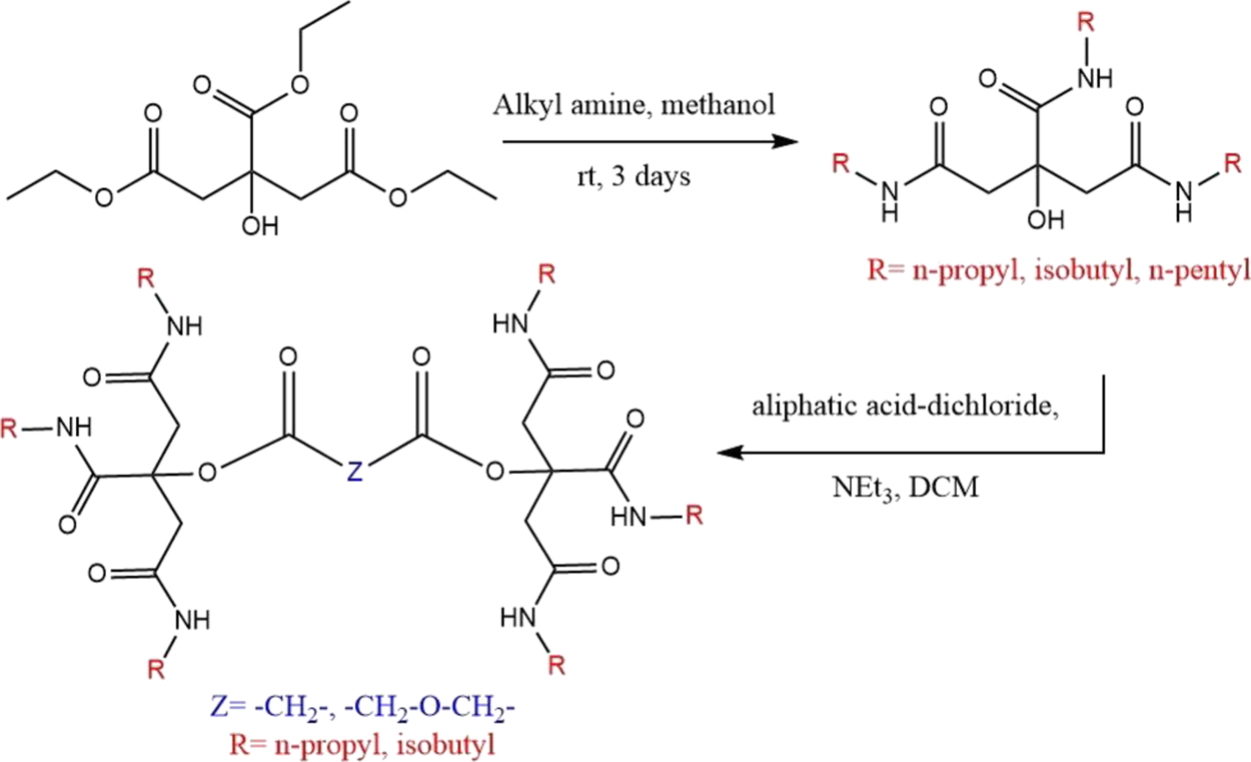
Synthetic scheme of the procedure adopted for the preparation of trialkyl citramides and bis(trialkyl citric acid) amides from triethyl citrate.

The synthetic procedure for trialkyl citramides and bis(trialkyl citric acid) amides is presented in Figure 9. Initially, triethylcitrate was reacted with alkyl amines to produce the trialkyl citramides. The formation of trialkyl citramides was confirmed by NMR spectroscopy.^21^ Trialkyl citramides were then reacted with aliphatic acid-dichlorides to yield bis(trialkyl citric acid) amides. Only two trialkylcitramides were made, which were soluble enough for KHI testing. They were tri-*n*-propylcitramide (TnPrCAm) and tri-iso-butylcitramide (TiBuCAm). It was previously known that the ethyl derivative was a poorer KHI, that the isopropyl derivative could not be made (probably for steric reasons), and that *n*-butyl or larger alkyl derivatives were insoluble in water.^21,22^ Tri-*n*-pentylcitramide was not tested as KHI and not reacted with any acid-dichlorides, due to its negligible solubility in water.

All the SCC KHI test results of trialkylcitramides, bis(trialkyl citric acid) amides, VP:VCap 1:1, and deionized water are summarized in Table 2 and in Table 3. PMA and PMA-trialkylcitramide esters are also included, but their synthesis and KHI performance are discussed later. In some SCC tests, solvent synergist (nBGE) or liquid hydrocarbon was also added.

**Table 2:** Slow Constant Cooling KHI Test Results for Mono- and Bis-(trialkyl citric acid) Amides

entry	sample	cloud p.t *T*_cl_/°C	conc. (ppm)	av. *T*_o_ (°C)	st. dev. (°C)	av. *T*_a_ (°C)
1	no additive			17.2	0.6	16.9
	1 mL of decane			16.0	0.4	15.7
	3 mL of decane			15.6	0.5	15.3
2	VP:VCap 1:1	85	2500	8.9	0.2	6.8
3	TnPrCAm	>95	2500	16.3	0.3	12.3
4	TiBuCAm	>95	2500	13.9	0.2	13.5
5	TnPeCAm	not soluble				
6	malonyl-TnPrCAm	>95	2500	16.3	0.3	13.5
7	malonyl-TiBuCAm	>95	2500	13.7	0.6	12.7
8	oxydiacetyl-TnPrCAm	>95	2500	12.8	0.2	12.3
9	oxydiacetyl-TiBuCAm	35	1000	9.3	0.05	9.2
			2500	8.4	0.1	8.1
10	oxydiacetyl-TiBuCAm + 1 mL of *n*-decane each cell		2500	7.4	0.1	7.2
11	oxydiacetyl-TiBuCAm + 1 mL of *n*-decane each cell + 5000 ppm nBGE		2500	8.1	0.2	7.9
12	oxydiacetyl-TiBuCAm + 3 mL of *n*-decane each cell + 5000 ppm nBGE		2500	6.2	0.2	6.0

**Table 3:** Slow Constant Cooling KHI Test Results for Polymaleic Trialkylcitramide Esters

entry	sample	conc. (ppm)	av. *T*_o_ °C	st. dev. °C	av. *T*_a_ °C
1	PMA (*M*_W_ 800)	2500	17.1	0.3	16.9
2	PMA-TnPrCAm	2500	13.4	0.6	13.1
3	PMA-TiBuCAm	2500	11.6	0.7	11.2
4	PMA-TiPrCAm/TiBuCAm (1:1)	2500	12.8	0.2	12.2

At least 5 (and up to 10) standard constant cooling tests were employed to get each average *T*_o_ and average *T*_a_ value, where *T*_o_ denotes the temperature at which the first macroscopic detectable gas hydrate is formed and *T*_a_ denotes the rapid hydrate formation temperature in a solution. The KHI performance of each citramide derivative was compared mostly based on the *T*_o_ values as complete avoidance of gas hydrate formation is the preferred goal for field operations. Thus, a lower *T*_o_ value of a solution corresponds to a better KHI performance. A significant difference between *T*_o_ and *T*_a_ values suggests a good ability of a KHI to arrest or delay catastrophic crystal growth for a longer period. However, caution in using this interpretation must be taken, since a low *T*_o_ value will mean a high driving force, which can cause more rapid macroscopic hydrate formation than a higher *T*_o_ value.

Deionized water and VP:VCap 1:1 (2500 ppm concentration) gave detected onset of hydrate formation at average *T*_o_ values of 17.2 and 8.9 °C, respectively. These data were used as references to gauge the KHI performance of the citramide derivatives. Table 2 shows that different citramide derivatives gave a wide range of KHI performance, which depends on the size of the alkyl groups in the citramide derivatives, the concentrations used, and the presence of additives. The size and shape of the hydrophobic groups are critical for optimal KHI performance. Previous research has shown that the iso-butyl group possesses a more optimal size and shape than the *n*-propyl group for some classes of KHI polymers as well as synergists. The branched tail of the iso-butyl group provides better interaction with the tertiary water structure for inhibiting nucleation and crystal growth.^20,30,31^

**Figure 10:**
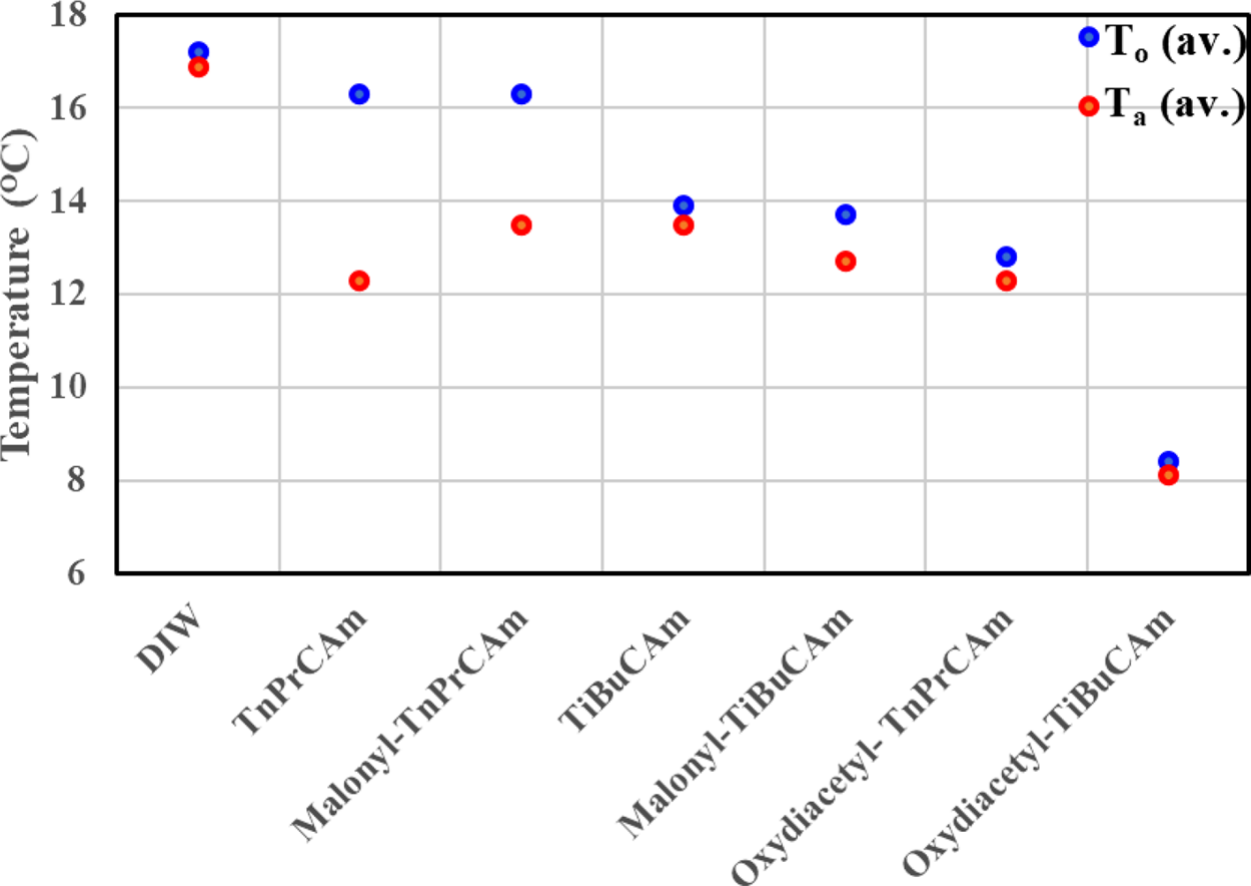
Graphical display of the KHI efficiency of different citramide derivatives with 2500 ppm concentration with respect to deionized water.

The expected poor KHI performance of TnPrCAm and improved performance of TiBuCAm were seen in the SCC rocking cell experiments. In Table 2, we can see that an aqueous solution of TnPrCAm having 2500 ppm concentration has an average *T*_o_ value of 16.3 °C, whereas TiBuCAm has a slightly better KHI performance with an average *T*_o_ value of about 13.9 °C under the same condition. However, both the citramides exhibited relatively poor KHI performance compared to commercial VP:VCap 1:1. This was expected due to the lack of sufficient hydrophobic groups, but they at least demonstrated the principle that KHI performance can be improved for a citramide derivative by increasing the hydrophobicity of the alkyl group (Figure 10). In a previous report of the structure–activity relationship analysis of poly(ethylene citramide)s, the KHI performance improved with the increasing size of the hydrophobic groups as long as water solubility was upheld through hydrogen bonding via the amide functional groups.^20^ Based on this knowledge, we attempted to test a more hydrophobic trialkylcitramide, but unfortunately tri-*n*-pentylcitramide was not water soluble.

We assumed that trialkylcitramides did not have enough hydrophobic groups for an optimal KHI effect. Therefore, bis(trialkyl citric acid) amides with six alkylamide groups were synthesized. Malonyl chloride and oxydiacetyl dichloride are the aliphatic acid-dichlorides that were used to yield the bis(trialkyl citric acid) amide derivatives. However, as Table 2 shows, we still obtained poor KHI performance with bis(trialkyl citric acid) amides where trialkylcitramides are connected through a malonyl group. For example, a 2500 ppm aqueous solution of malonyl-TnPrCAm gave an average *T*_o_ value of 16.3 °C, which is the same as that of TnPrCAm. A 2500 ppm aqueous solution of malonyl-TiBuCAm gave an average *T*_o_ value of about 13.7 °C, which again is almost the same as the monocitramide, TiBuCAm. However, the KHI test results were improved for biscitramides made from oxydiacetyl dichloride. Thus, 2500 ppm oxydiacetyl-TnPrCAm gave better KHI performance than TnPrCAm with the average *T*_o_ value dropping from 16.3 to 12.8 °C. Polymers with *n*-propyl groups are known to be good KHIs, so these results suggest that use of six *n*-propylamide groups is insufficient to obtain a good KHI good performance for bis(tri-*n*-propyl citramide)s. This gave an indication that the use of an extra −CH_2_O– group on the aliphatic acid-dichloride was advantageous for improved KHI performance of oxydiacetyl-TnPrCAm compared to malonyl-TnPrCAm (Figure 10).

Oxydiacetyl-TiBuCAm made by connecting two molecules of TiBuCAm through an oxydiacetyl group was found to be the most effective citramide derivative investigated in this study. An aqueous solution of oxydiacetyl-TiBuCAm having 2500 ppm concentration gave an average *T*_o_ value of 8.4 °C (Table 2). This compares to 8.9 °C for the VP:VCap 1:1 copolymer. In terms of the structure–activity relationship, the improved performance for oxydiacetyl-TiBuCAm can be compared to the following:

(a) TiBuCAm – increased number of alkylamide groups from 3 to 6.

(b) Oxydiacetyl-TnPrCAm – increased alkyl size with end-branching.

(c) Malonyl-TiBuCAm – increased -CH_2_-O-CH_2_- spacer group compared to -CH_2_-.

For this last factor, we presume that the larger spacer group giving more distance and flexibility of the two triisobutylcitramide groups is important for the KHI performance.

At a concentration of 2500 ppm, none of the citramide derivatives except oxydiacetyl-TiBuCAm (*T*_cl_ = 35 °C) showed a cloud point when heated up to 95 °C. No sign of turbidity was observed for other citramide derivatives at any stage of the heating process up to this temperature. A lower cloud point, near the hydrate formation temperature, has been shown to improve the performance of various classes of polyamide as long as the optimum size, shape, and density of pendant alkylamide groups are maintained while still retaining water solubility.^32^ Possible reasons include

1. Maximizing the polymer surface area/hydrodynamic volume (or weight) ratio
2. Maximizing the hydrophobic interactions of the polymer at a temperature when hydrogen bonding is about to break down
3. Greater concentration at interfacial regions (gas–water or oil–water) where hydrate formation first occurs.

None of the monocitramides or biscitramides or maleic-citramide esters showed good gas hydrate crystal growth inhibition as judged by the small difference between the *T*_o_ and *T*_a_ values for all new molecules. This can be compared to the VP:VCap 1:1 copolymer, which is from the family of *N*-vinyl lactam polymers, which are known to be good for arresting hydrate crystal growth.^15,33−37^ The main drawback of poor crystal growth retardation is that if hydrate nucleation does ever occur in a flow line, macroscopic hydrate formation will be fast and the operator has little time to react to prevent build-up of hydrate deposits. Therefore, the operator must ensure injection of the correct KHI dosage to avoid such a situation.

We then investigated the effect of an artificial liquid hydrocarbon phase and also an external synergist on the KHI performance of our best citramide derivative. Even on natural gas fields, there is usually some associated liquid hydrocarbon, and if the KHI components partition to this phase, this can lead to loss of KHI performance. We added 1 mL of *n*-decane as a liquid hydrocarbon to each cell of 20 mL of 2500 ppm aqueous solution of oxydiacetyl-TiBuCAm before the KHI experiment. The result was that the average *T*_o_ value dropped from 8.4 °C without *n*-decane to 7.4 °C with *n*-decane. We presume that the better KHI performance is due to lowering of the system equilibrium temperature by addition of *n*-decane but also indicates that the citramide does not partition significantly to the hydrocarbon phase.^38^ Also, 1 mL of decane was added to the cells, and the subcooling temperature was approximately 15.5 °C.^39^

nBGE has been shown to be a good solvent synergist to improve the efficiency of many KHI polymers.^40−43^ Therefore, we added 5000 ppm of nBGE as a solvent synergist in *n*-decane containing oxydiacetyl-TiBuCAm solution (2500 ppm) to improve further its KHI performance. The results showed that nBGE had a weak negative result on the KHI efficiency of oxydiacetyl-TiBuCAm. Possibly, the *n*-butyl group of the solvent synergist is in competition with the isobutyl group of citramide, which may cause an overall negative effect of nBGE. However, addition of further 2 mL of *n*-decane (total 3 mL) to that solution helped to give an improved KHI performance, lowering the average *T*_o_ value from 8.1 to 6.2 °C. This could be partially due to an additional lowering of the equilibrium temperature.^39^

**Figure 11:**
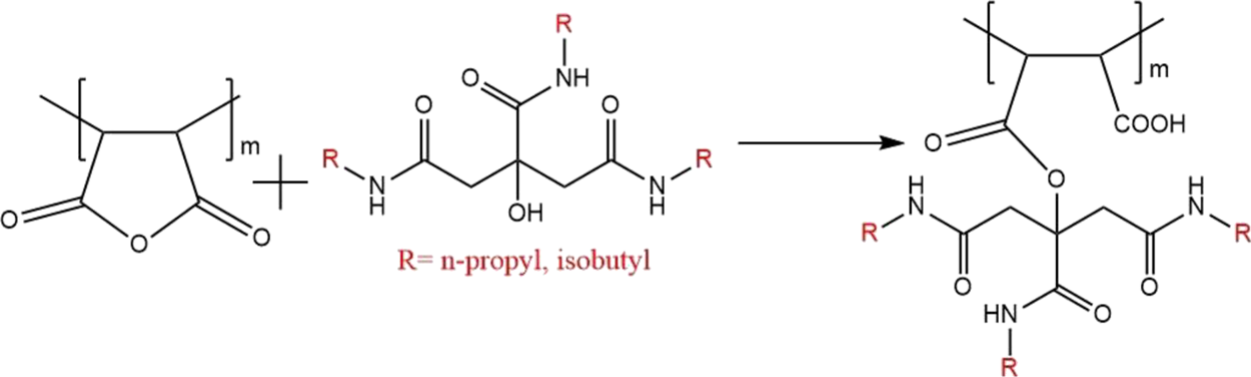
Synthetic scheme of the procedure adopted for the preparation of polymaleic trialkylcitramide esters from polymaleic anhydride.

Using another approach to increase the number of citramide units, we decided to ring-open the anhydride units of PMA with trialkylcitramides to make polymaleic trialkylcitramide esters (Figure 11). The maleic anhydride units of PMA react slowly with water or the hydroxy group of trialkylcitramides to give maleic acid groups and or ester groups. Thus, when making 2500 ppm solutions of PMA, all anhydride units are converted to acid groups. The SCC results are given in Table 3. PMA itself showed a negligible KHI effect and an average *T*_o_ value of about 17.1 °C, which is close to the observed gas hydrate formation with no additive. This is undoubtedly due to a complete lack of pendant hydrophobic groups as observed in other classes of polyacids, polyols, or polyamides.^19,44^ Therefore, we functionalized PMA with trialkylcitramides by reacting with the anhydride units. The results in Table 2 show that PMA-TnPrCAm exhibited some improved KHI effect compared to pure PMA with an average *T*_o_ of 13.4 °C. The KHI performance was further improved by functionalizing PMA with TiBuCAm with an average *T*_o_ of 11.6 °C. In contrast, introducing both TnPrCAm and TiBuCAm in a 1:1 ratio on PMA showed an intermediate KHI efficiency. In summary, we did not obtain as good KHI performance with the maleic-citramide ester polymers as the best bis-citramide, oxydiacetyl-TiBuCAm. The high percentage of carboxylic acid groups (50% of all pendant functional groups) making the maleic ester polymers much more hydrophilic than oxydiacetyl-TiBuCAm with its low cloud point is probably a critical factor.

## 4 Conclusions

A series of citramide molecules with varying size and shape and number of hydrophobic groups has been synthesized and investigated as KHI in high-pressure rocking cells against an sII hydrate-forming gas mixture under 76 bar using the SCC test method. Increasing the size of alkyl substitutes was found to be an important factor to give better KHI performance as long as the water solubility was maintained. The KHI performance of citramide derivatives was significantly improved by either converting monotrialkylcitramide (three alkylamide groups) to bis(trialkyl citric acid) amides (six alkylamide groups) or attaching the citramide to the polymer PMA. However, the distance between the trialkylcitramide groups appears to be critical for good performance. The best molecule was found to be a bis(tributyl citric acid) amide with an oxydiacetyl group as a linker between the two citramides (oxydiacetyl-TiBuCAm). It performed better than a low *M*_W_ VP:VCap copolymer. None of the synthesized citramide derivatives showed a strong ability to retard the catastrophic crystal growth once nucleation had started, indicating that the main mechanism operating is nucleation inhibition. To mimic the presence of a liquid hydrocarbon phase, KHI tests with *n*-decane were carried out. This lead to lower onset temperatures for oxydiacetyl-TiBuCAm, at least partly due to lowering of the hydrate system equilibrium. In summary, we have demonstrated that good KHI performance can be obtained with molecules with as little as six alkylamide groups but worse for three groups. Whether the performance is optimized for this number of alkylamide groups depends on the geometry, the size of alkyl substituents, and water solubility.^45^
